# Assessment of psychometric properties of the modified experiences of teaching–learning questionnaire in Iranian nursing students

**DOI:** 10.1186/s12909-022-03365-z

**Published:** 2022-04-25

**Authors:** Mohammadali Hosseini, Amir Jalali, Nader Salari

**Affiliations:** 1 Department of Medical Education, Virtue University of Medical Sciences, Tehran, Iran; 2 Nursing Department, University of Social Welfare and Rehabilitation Sciences, Tehran, Iran; 3 Substance Abuse Prevention Research Center, Research Institute for Health, Kermanshah University of Medical Sciences, Kermanshah, Iran; 4 Department of Biostatistics, Faculty of Public Health, Kermanshah University of Medical Sciences, Kermanshah, Iran

**Keywords:** Reliability, Validity, Teaching–learning experiences, Persian

## Abstract

**Background:**

Universities are in charge of training, educating, and preparing students for their future. Teaching and learning methods have a profound role in fulfilling this responsibility by universities. Examining teaching–learning experiences needs a proper tool to collect the information needed. The aim of this study was to collect validity evidence of the modified experiences of teaching–learning questionnaire (ETLQ) in Iranian nursing students.

**Methods:**

The validation process was started by securing the required permissions from the designer of the tool. Then the tool was translated into Farsi using forward–backward method. After preparing a Farsi version of the tool, the content, response process, and internal structure assessment were checked and supported using qualified methods. To examine internal structure, Exploratory Factor Analysis (EFA) and Confirmatory Factor Analysis (CFA) were conducted for three sections of the scale with the participation of 278 nursing students. To examine the reliability of the tool, test–retest method was used and internal correlation was examined using Cronbach’s alpha.

**Results:**

The EFA and CFA results confirmed the tool with three domains, seven factors and 33 items. The R^2^-index of the model was obtained equal to 0.99, which indicates that 99% of the changes in teaching–learning experiences are explained by the tool (33 items). The main indices in CFA were higher than 0.9, which indicates the goodness of fit of the model. Pearson correlation between the items and the subscales was significantly and directly related to the whole scale. Moreover, with Cronbach’s alpha equal to 0.944 and test–retest result equal to 0.88, reliability of the Farsi version of modified ETLQ was supported.

**Conclusion:**

The results showed that the Farsi version of modified ETLQ had acceptable and applied indices to measure teaching–learning experiences in nursing students. The tool can be used as a valid tool in different fields of education in medical sciences.

**Supplementary Information:**

The online version contains supplementary material available at 10.1186/s12909-022-03365-z.

## Introduction

Continuous examination of the methods and processes of improving and developing learning performance in students is one of the major measures and educational institutes and universities have conducted several studies in this field [1, 2]. The most important part of the examination is to use students’ feedback about learning and teaching processes [3]. Several studies have highlighted the importance of the relationship between students’ learning and their experiences in learning and teaching environments [1]. Examining teaching–learning experiences is one of the efficient ways to develop learning in students and it requires using the information provided by students [4]. In addition, the elements of teaching and learning environment [5] such as the teachers, emotional environment, personal specifications of students [6], teacher-student emotional interactions [7], teaching–learning experiences of students, and their feedback [1] are the key factors that can improve the quality of education through efficient programming and coordination [2].

Several studies have been carried out in this field in different fields of study [4, 7, 8]. Netshifhefhe et al. argued that improving the quality of teaching–learning process, guaranteeing the quality of teaching, and examining the learning process are highly imperative [9]. The teachers’ knowledge and skills are formed through interacting with learners[10]. Efficient interactions between instructor and learner and receiving feedback from learners in the teaching–learning process are key factors in the development of knowledge and skills in instructors[2].

Given the above introduction, the education environment is a key factor in motivating learning as it boosts positive behaviors in line with learning and eventuates in educational achievement [11]. Valuable information is attainable through assessing students’ experiences with the learning environment and their experiences with the teaching–learning process in particular. Such information can be used to develop efficient programs and improve learning methods [12]. Diverse tools are available to examine the feedback and experiences of students with a learning environment and teaching – learning process in particular [1, 13, 14]. One of these tools is Entwistle et al.’s (2003) experience of teaching–learning questionnaire (ETLQ), which was first developed in English [12]. The tool was further developed in Finland and introduced as two modified questionnaires [1]. Eventually, the tool was modified further by Utriainen et al. (2018) with three domains of teaching–learning environment, students’ approach to learning, and critical thinking with eight factors, three sub-factors, and 33 items. This tool is valid and reliable in Finland [14] and it is very important to use valid and reliable tools to assess the learning and educational status [15]. The use of valid and reliable tools in studies creates confidence in the results of the study. In addition, due to limited resources and the great deal of time and money spent on research, it is important to make sure that the tools used are valid, reliable, and trustworthy [15, 16].

Taking into account the absence of a valid and reliable tool to measure teaching–learning experiences in Iran, the present study is an attempt to measure the psychometrics of Utriainen’s et al. questionnaire with 33 items [14]. Therefore, the aim of this study was to collect validity evidences of the modified experiences of teaching–learning questionnaire (ETLQ) in Iranian nursing students.

## Methods

### Modified experiences teaching learning questionnaire

The ETLQ was introduced by Entwistle et al. (2003) in Amsterdam- Netherlands [12]. The tool was further developed by Parpala et al. (2013) in Finland as two modified questionnaires [1]. The tool was also modified by Utriainen et al. (2018) with 33 items, 11 factors and three sections of teaching–learning environment with four factors (alignment, peers’ support, constructive feedback, and encourage to learn with three sub-factors of disciplinary understanding, teaching for perception, and supportive teaching). The second subscale is students’ approach to learning with three factors (deep approach, surface approach, and organized studying) and the third subscale is critical thinking skills (Table 1). The items are designed based on Likert’s five-point scale (agree = 1, …, disagree = 5) [14]. The tool has been validated in other countries as well [1, 14]. Validity coefficient of the subscales and factors is listed in Table 1 [14].

**Table 1:** The modified ETLQ scale and its subscales

Aspects	Factors	Sub-factors	Items	Cronbach's Alpha
Teaching–learning environment	Alignment		**8,16,17,18**	**0.74–0.78**
	Peer support		**13,14,15**	**0.73–0.74**
	Constructive feedback		**10,11,19**	**0.82–086**
	Encouraging learning	Disciplinary understanding	**4,6**	**0.67–0.69**
		Teaching for understanding	**1,2,5,12**	**0.77–0.79**
		Supportive teaching	**7,9**	**0.64–0.65**
Approaches to learning	Deep approach		**23,24,25,26,29**	**0.73–0.78**
	Surface approach		**20,21,22**	**0.63**
	Organized studying		**27,28**	**0.72–0.73**
Critical thinking skills			**30,31,32,33**	**0.8–0.81**

### Design and sitting

**Fig. 1:**
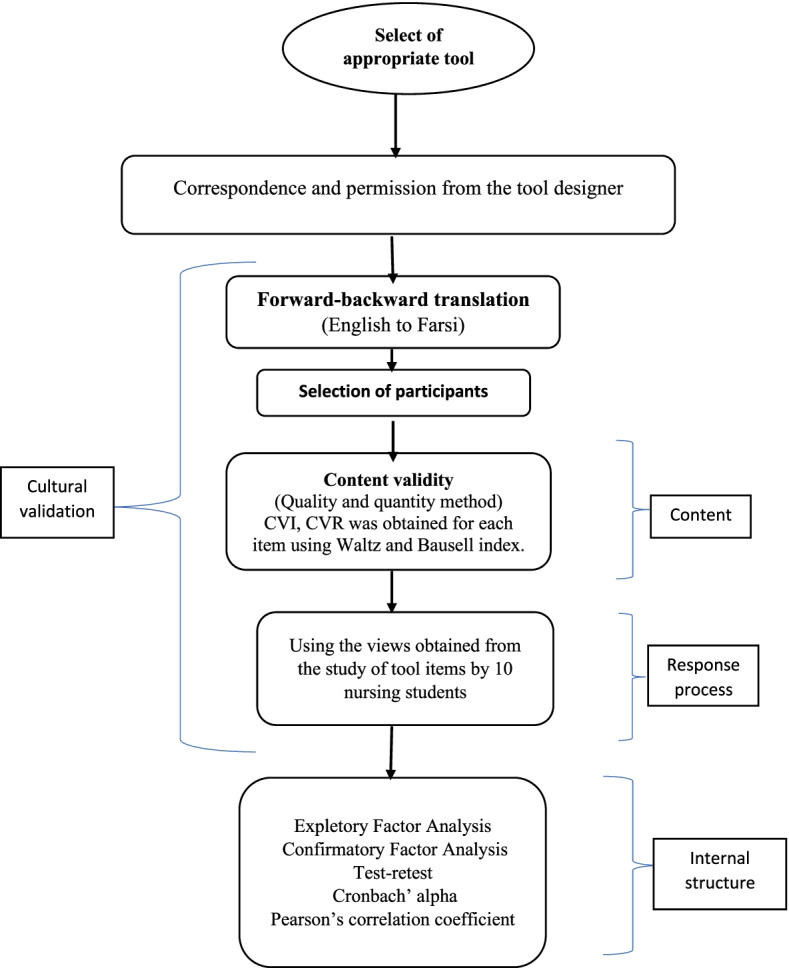
Diagram of research process

A methodological and validation study was carried out on a study population of 2^nd^, 3^rd^, and 4^th^ years nursing students at Kermanshah University of Medical Sciences. Totally, 278 students were selected through convenient sampling. The questionnaire used in this study was modified ETLQ designed and modified by Utriainen et al. (2018). The questionnaire has three sections namely teaching–learning, students’ attitude to learning, and critical thinking with 11 factors, three sub-factors, and 33 items (14). Before translating the tool, the designer of the tool was contacted and required permissions were taken. In this post-translational study, the evidence gathering process required for the validity of the instrument was performed based on the cultural validation (17) model and using the three steps of content evaluation, response process, and internal structure (Fig. 1) (15, 16).

### Cultural validation

Cultural validation was done using Wild et al.’s method (17) and then the tool was translated using forward–backward method by two independent translators from English into Farsi. The translated works were then backward translated into English by two other independent translators. After examining the translated tools by researchers and experts, a final Farsi version was obtained. Then as pretest, the final translation was provided to some of the students from the study population to highlight probable issues if any. This stage led to no alteration in the tool. Afterwards, the tool was edited by an expert in Farsi literature. After documenting the whole process, the final version of the tool was used for psychometric examinations (validity and reliability).

### Participants

The study population consisted of undergraduate nursing students of 2^nd^, 3^rd^, and 4^th^ years and postgraduate nursing students (MSc) of 1^nd^, 2^nd^ years. In qualitative and quantitative content assessment phase, the tool was distributed among 20 faculty board members and experts in nursing and health education (12 questionnaires were returned). In the response process, 10 students filled out the tool and in test–retest phase, 20 students filled out the tool. In the internal structure phase, 530 students were selected; in the first stage, 170 questionnaires were examined and exploratory factor analysis was performed. Then the number of questionnaires reached 278 questionnaires, which were used in the confirmatory factor analysis stage and 252 partially filled-out questionnaires were excluded.

### Data analysis

#### Content

As to content validity, 12 experts and faculty board members from different fields were asked to examine the tool (qualitative content validity). To determine content validity quantitatively, a content validity index was obtained for each item using Waltz and Bausell index.

#### Internal structure

To examine the reliability of the tool, a test–retest method was used and internal consistency was examined using Cronbach’s alpha. In addition, EFA and CFA were used for validation of the tools. As to internal structure, each one of the three main parts of the questionnaire was examined using EFA and CFA. Then EFA and CFA were performed for the modified ETLQ (three sections namely teaching–learning environment, students’ approach to learning, and critical thinking) as the main questionnaire. All data analyses were done in SPSS (v.18) and LISREL (v.8).

#### Response process

In the present study, the response process was performed qualitatively and using the viewpoints of 10 nursing students.

## Results

### Descriptive results

The modified ETLQ was validated with the participation of 278 nursing students (170 students for EFA and 278 students for CFA). In the case of EFA, 53.5% of the students were female and 42.9% were male. The mean age of the 170 students was 25.28 ± 4.66 with minimum and maximum ages equal to 20 and 46 years respectively. In the case of CFA, 52.9% of the students were female and 47.19% were male. The mean age of 278 students was 25.32 ± 4.43 with minimum and maximum ages equal to 20 and 46 years respectively. The rest of the demographics are listed in Table 2.

**Table 2:** Demographic characters of participants in study

Variables		N (%)	
		**EFA**	**CFA**
Gender	Female	91(53.5)	147(52.9)
	Male	79(46.5)	131(47.1)
Marital Status	Unmarried	126(74.1)	205(73.7)
	Married	44(25.9)	73(26.3)
Grade	Bachelor	114(67.1)	176(63.3)
	Master of Science	56(32.9)	102(36.7)
Housing	Non-dormitory	94(55.3)	153(55)
	Dormitory	76(44.7)	125(45)

As the findings showed, 52.9% of the participants were women, 73.7% were unmarried, 63.3% were undergraduate students, and 55% were of the local population.

### Content and response process

Content assessment was measured using two methods. In the qualitative method, the questionnaire was examined in terms of the layout of the items, and relevance to the objectives. Through a quantitative method, 12 experts took part in the study and the CVR of the tool was obtained equal to 0.8 and in the 0.58–0.92 range. In addition, the CVI of the tool was equal to 0.85, which is in the 0.75–1 range. The observed skewness for all statements ranged from -0.86 to 0.16 and the value of Kurtosis was from -1.05 to 0.83, which was within (-2, 2) interval. This means the distribution of the statements is approximately symmetrical (Table 3).

**Table 3:** The modified ETLQ and results of CVI, CVR, Skewness and Kurtosis

No	Items	CVI^a^	CVR^b^	Skewness^c^	Kurtosis^d^
1	This education program encourages me to relate to the problems of the wider world with what I am learning	0.83	0.75	-0.45	-0.2
2	I can understand the importance of what we are taught in this program	0.75	0.67	-0.49	-0.06
3	I enjoy being in touch with this training program	0.83	0.75	-0.36	-0.56
4	Instructors help us communicate, think, and come to terms with issues	0.92	0.83	-0.52	-0.43
5	Teaching this curriculum helps me to think about the various manifestations of the basic evidence in this field	0.75	0.83	-0.44	-0.29
6	This training program teaches me how to uncover the hidden features in the topics of the curriculum	0.83	0.92	-0.38	-.036
7	Instructors try to share their passion with us in this training program	0.92	0.75	-0.29	-.036
8	How to teach this curriculum is well coordinated with what I have to learn	1	0.75	-0.27	-0.47
9	Instructors are patient in explaining what seems difficult to students	1	0.83	-0.38	-0.46
10	The feedback I give on my assignments helps me improve my learning and study methods	0.83	0.67	-0.2	-0.35
11	The feedback given to my entire assignments helps to clarify topics that I do not understand well	0.75	0.92	-0.27	-0.39
12	The set of assignments helps me to connect my knowledge and experiences	0.83	0.92	-0.41	-0.49
13	I feel that in general I can work with other students in this course	0.92	0.83	-0.68	0.28
14	In this training program, students support each other and help each other if needed	0.75	0.92	-0.82	0.38
15	Talking and thinking with other students helps develop my understanding	0.75	0.75	-0.73	-0.002
16	The expectations of the instructors in evaluating the assignments of this training program are clear to me	0.92	0.67	-0.38	-0.53
17	I can see how the set of assignments helps me learn	0.75	0.67	-0.54	-0.36
18	What I am going to learn in this training program is clear to me	0.83	0.58	-0.46	-0.25
19	I regularly get feedback from professors about my educational status	0.92	0.75	-0.39	-0.4
20	In general, I act in a regular and planned way during my studies	0.92	0.75	-0.35	-0.33
21	I usually put a lot of effort into reading	0.75	0.83	-0.55	0.06
22	In this training program, I carefully organize my study time to make the most of it	0.83	0.92	-0.6	-0.26
23	Before concluding, I look carefully at the evidences from what I read	0.83	0.92	0.16	0.48
24	The ideas I get from studying the curriculum often led me to long chains of thinking	0.75	0.83	-0.53	-0.32
25	When I connect with my ideas, I can think about how to achieve good results	0.92	0.83	-0.4	-0.22
26	If I do not understand the issues well while studying, I will try a different approach	0.92	0.75	-0.17	-0.3
27	I often have trouble understanding the things I need to remember	0.83	0.67	-0.34	-0.6
28	Most of what I learn seems to be nothing more than a lot of irrelevant bits and pieces in my mind	0.83	0.83	-0.67	0.4
29	I usually put concepts together to understand the meanings of what we need to learn	0.92	0.92	-0.72	0.18
30	In this training program, I learn to always use the process of analyzing and organizing information	0.92	0.92	0.15	-1.05
31	I learn to critically evaluate issues	0.92	0.75	-0.86	0.83
32	I learn to use theoretical knowledge well in exercises	0.92	0.92	-0.37	0.16
33	I learn to develop new ideas	0.92	0.92	-0.65	-0.36

^a^Content Validity Ratio, ^b^Content Validity Index, ^c^Skewness is a measure of symmetry, or more precisely, the lack of symmetry, ^d^Kurtosis is a measure of whether the data are heavy-tailed or light-tailed relative to a normal distribution

To determine the response process, a qualitative approach was followed and the tool was examined in terms of the fluency and understandability of the items, grammar, and wording. To this end, 10 nursing students commented on the tool.

### Internal structure

#### Exploratory Factor Analysis (EFA)

Before performing EFA, correlation coefficients of the items for three sections of the scale (teaching–learning environment, students’ approach to learning and critical thinking) and modified ETLQ were checked. To this end, KMO and Bartlett’s test of sphericity were used (Table 4). With KMO equal to 0.865, the correlation between the data for factor analysis was confirmed. In addition, Bartlett’s test results supported EFA.

**Table 4:** The KMO and Bartlett’s test of sphericity in Modified ETLQ and the sections

Scales and the section	KMO test	Bartlett’s test of sphericity		
		Chi-square	DF	*P*_value_
teaching–learning environment (Sect. 1)	0.911	1824.513	171	0.0001
Approach to learning (Sect. 2)	0.89	836.391	45	0.0001
Critical thinking (Sect. 3)	0.64	150.624	6	0.0001
Modified ETLQ	0.887	3739.325	528	0.0001

The EFA was performed on the19 items (Section-one as teaching–learning environment), 10 items (section- two as students’ approach to learning), and 33 items of the modified ETLQ and the factors were extracted using Principal Components and Varimax rotation methods.

**Fig. 2:**
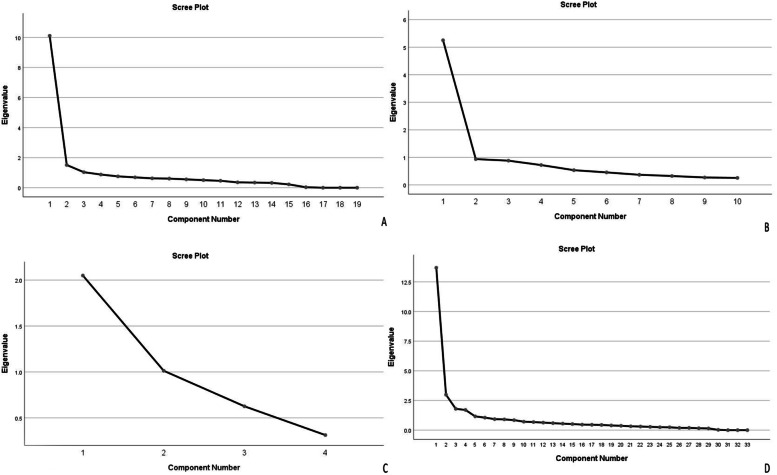
**A** Scree Cattel plot of the extracted elements of the section- one modified ETLQ, **B** Scree Cattel plot of the extracted elements of the section- two modified ETLQ and **C** Scree Cattel plot of the extracted elements of the section- three modified ETLQ, **D** Scree Cattel plot of the extracted elements of the modified ETLQ

To determine the number of factors, those with a specific value > 2 was selected. The primary results supported four factors in section one, two factors in section two, and one factor in section three for modified ETLQ. In addition, three sections were identified in modified ETLQ. Complementary Tables 1, 2, 3 & 4 list the extracted factors along with specific values, share of each factor in the three sections of modified ETLQ (section one with 19 items and four factors; section two with 10 items and two factors; and section three with 4 items and one factor) and the share of the three sections of the main questionnaire (33 items and three sections or main factor) along with accumulated variance. The scree plot generated in SPSS shows that the factors or elements can be used for the final analysis (Fig. 2).

Table 5 lists the rotated factor analysis in which the items with factor load higher than 0.3 are loaded on the related element. As listed, the EFA yielded three factors and 33 items. The extracted factors, items, and Cronbach’s alpha are listed in Table 5.

**Table 5:**
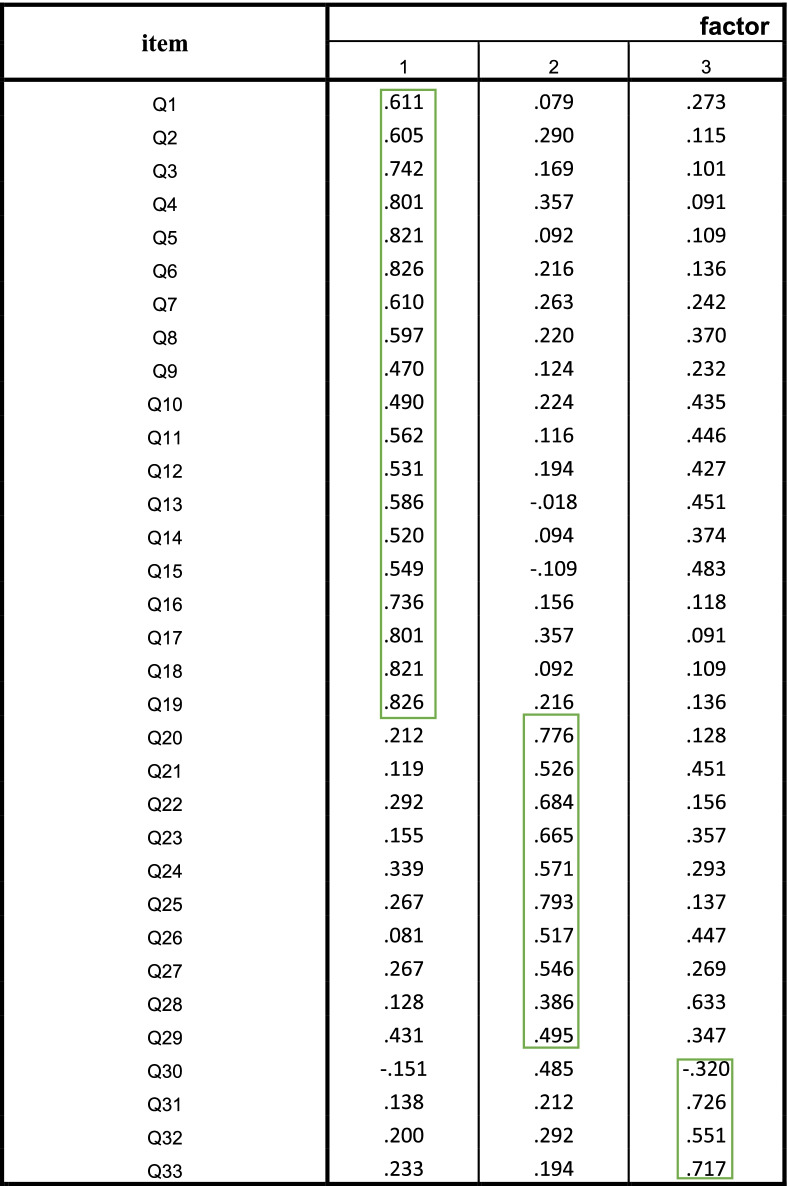
Matrix of factor loads of modified ETLQ questions on components after rotation

Extraction Method: Principal Component Analysis

Rotation Method: Varimax with Kaiser Normalization

a. Rotation converged in 7 iterations

#### Confirmatory Factor Analysis (CFA)

To examine validity of the model, the CFA method was used for modified ETLQ and its three sections and to this end, factor loads of each item was taken into account. With t-value > 1.96 in CFA, the level of significance is equal to 0.05 with a level of confidence equal to 95%. Moreover, with t-values higher than 2.576 and 3.29, the level of significance is equal to 0.01 (99%) and 0.001 (99.9%) respectively.

**Fig. 3:**
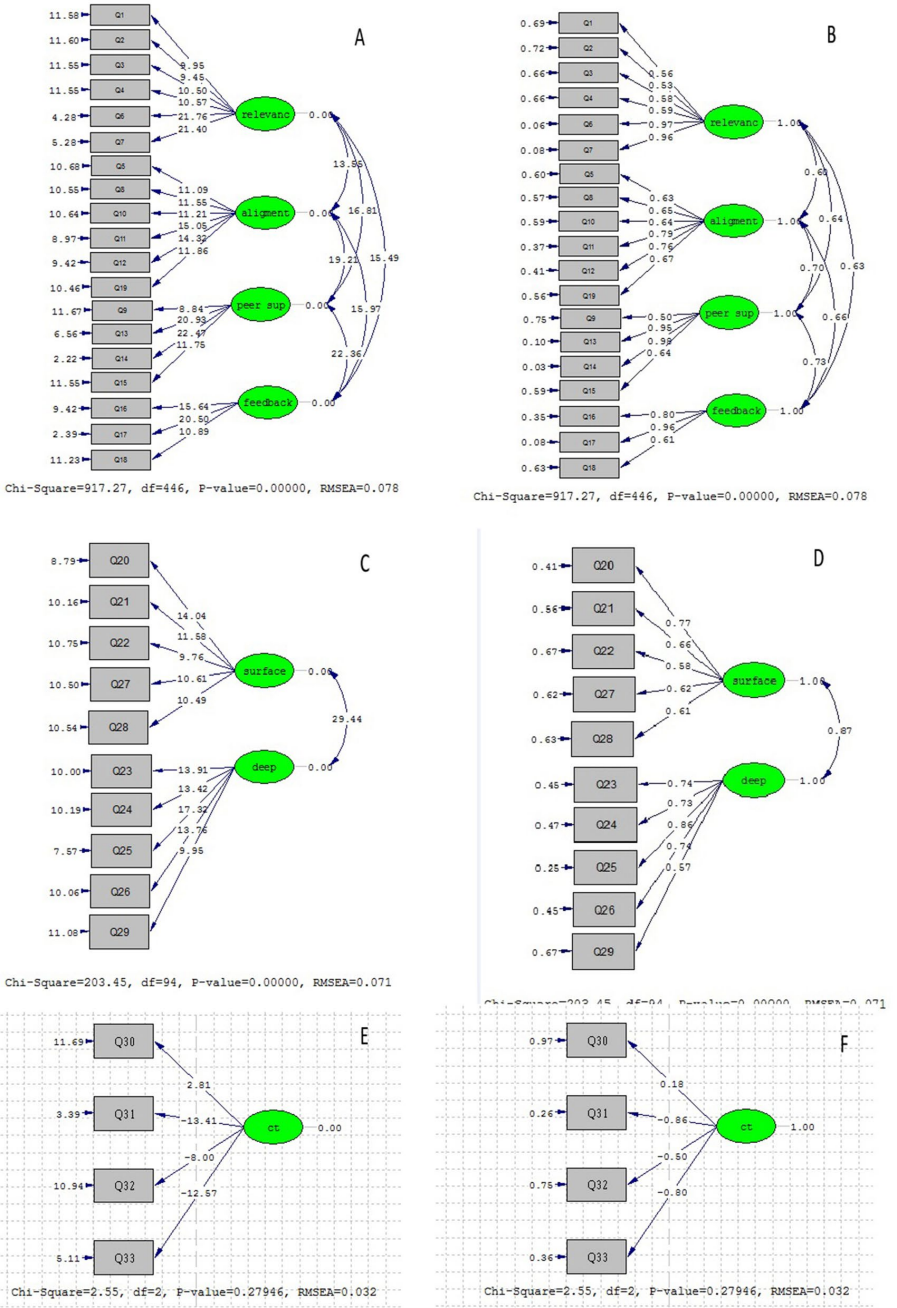
Four factor model of the Teaching learning environment in Iranian nursing students (**A**. significant state **B**. Standard), Two factor model of the Approach to learning in Iranian nursing students (**C**. significant state **D**. Standard), one factor model of the critical thinking in Iranian nursing students (**E**. significant state **F**. Standard)

**Fig. 4:**
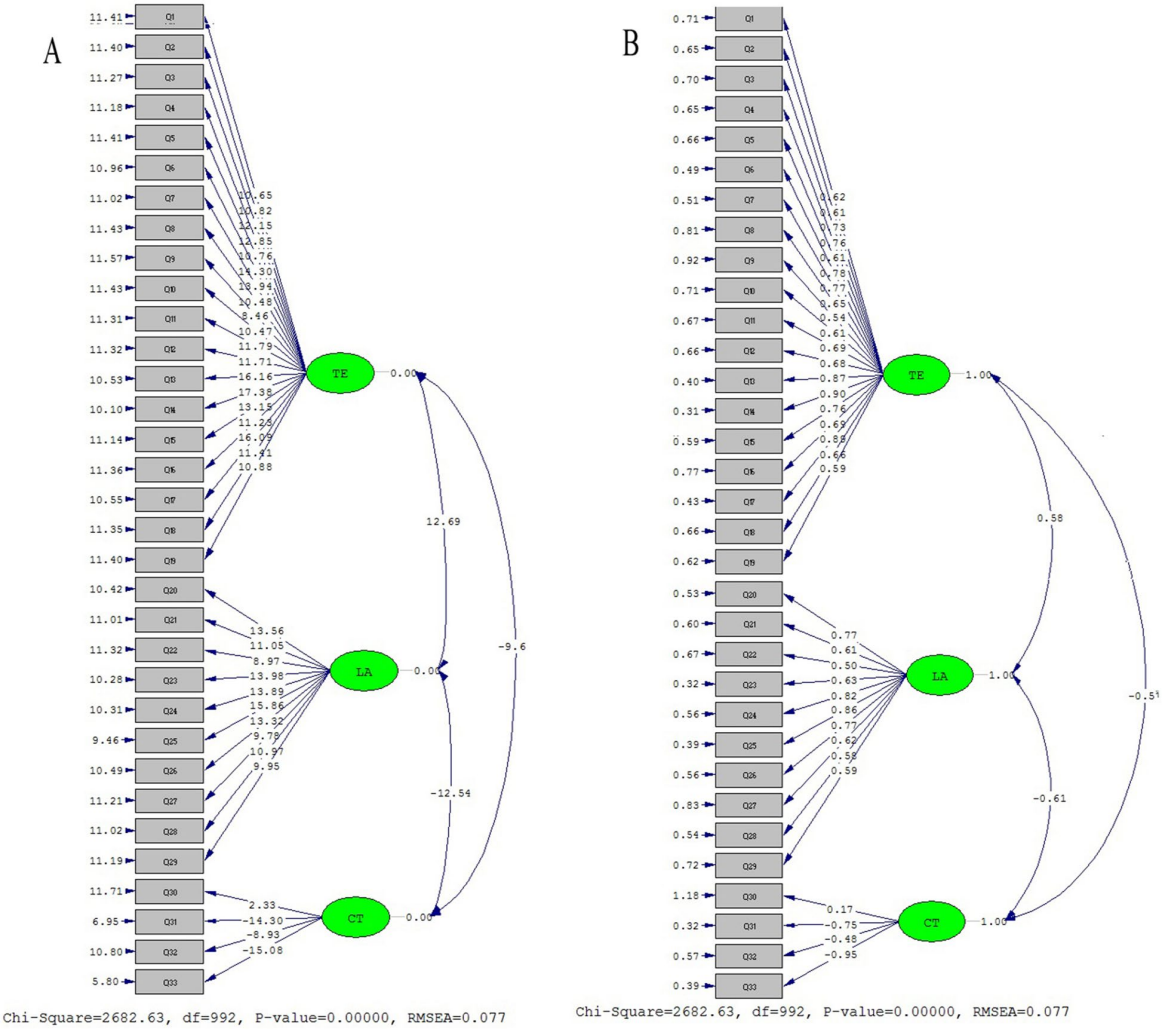
Three section model of the modified ETLQ in Iranian nursing students (**A** significant state), (**B** standard state)

The results of CFA showed three sections, seven factors and 33 items. Table 6 and Figs. 3 and 4a, b illustrate the results of CFA in two modes of significant (a) and standard (b) coefficients. Given the results and that all the figures are higher than 1.96, none of the items were eliminated.

**Table 6:** T-value Pearson correlation coefficient and factor loadings of the tool items

Factor	No	t _value_^a^	^b^ (λ)	R^c^	Sig
Teaching–learning environment (TL)	1	10.62	0.62^***^	0.624	0.0001
	2	10.82	0.61^***^	0.632	0.0001
	3	12.15	0.73^***^	0.719	0.0001
	4	12.85	0.76^***^	0.833	0.0001
	5	10.76	0.61^***^	0.755	0.0001
	6	14.30	0.78^***^	0.83	0.0001
	7	13.94	0.77^***^	0.69	0.0001
	8	10.48	0.67^***^	0.722	0.0001
	9	8.46	0.54^***^	0.52	0.0001
	10	10.47	0.61^***^	0.63	0.0001
	11	11.79	0.69^***^	0.688	0.0001
	12	11.71	0.68^***^	0.688	0.0001
	13	16.16	0.87^***^	0.646	0.0001
	14	17.38	0.90^***^	0.633	0.0001
	15	13.15	0.76^***^	0.631	0.0001
	16	11.23	0.69^***^	0.716	0.0001
	17	16.06	0.88^***^	0.807	0.0001
	18	11.41	0.66^***^	0.738	0.0001
	19	10.88	0.59^***^	0.786	0.0001
Approaches to learning (LA)	20	13.56	0.77^***^	0.762	0.0001
	21	11.05	0.61^***^	0.693	0.0001
	22	8.97	0.50^***^	0.719	0.0001
	23	13.98	0.63^***^	0.739	0.0001
	24	13.89	0.82^***^	0.775	0.0001
	25	15.86	0.86^***^	0.812	0.0001
	26	13.32	0.77^***^	0.6	0.0001
	27	9.78	0.62^***^	0.651	0.0001
	28	10.97	0.58^***^	0.674	0.0001
	29	9.95	0.59^***^	0.715	0.0001
Critical thinking skills (CT)	30	2.33	0.17^*^	0.286	0.0001
	31	-14.30	-0.75^***^	0.771	0.0001
	32	-8.93	-0.45^***^	0.68	0.0001
	33	-15.08	-.095^***^	0.766	0.0001

^***^P < 0/001; **P < 0/01; * *P* < 0/05

^a^The calculated values for all factor loadings of the first and second orders are greater than 1.96 and are therefore significant at the 95% confidence level, ^b^The specific value, which is denoted by the Lamda coefficient and the statistical symbol λ, is calculated from the sum of the factors of the factor loads related to all the variables of that factor, ^c^Pearson Correlation coefficient

In addition, Table 7 lists the goodness of fit indices in CFA model (modified ETLQ and its sections). Given the indices, the goodness of the fit of the model is supported.

**Table 7:** Fit Indicators Confirmatory Factor Analysis Persian modified version of ETLQ and its sections

Fit indicators	χ2/DF	CFI	NNFI/TLI	AGFI	RMSEA
Modified ETLQ and its sections					
Teaching–learning environment (Sect. 1)	2.05	0.91	0.91	0.81	0.074
Approach to learning (Sect. 2)	2.15	0.9	0.9	0.8	0.071
Critical thinking (Sect. 3)	1.27	0.99	1	0.98	0.032
Modified ETLQ	2.65	0.94	0.93	0.82	0.064
**Criterion**	≥ 3	< 0.9	< 0.9	< 0.8	> 0.08

Reliability of the tool was obtained using a test–retest method equal to 0.88 using the data collected from 20 nursing students in two phases with a 14-day interval.

To examine internal reliability of the items, Pearson’s correlation coefficient was used, which showed a direct and significant correlation between the items and the whole scale (Table 6). In addition, there was a direct and significant correlation (< 0.0001) between the subscales of ETLQ and the total score of the scale (Table 8).

**Table 8:** Correlation between research model factors

Pearson Correlation coefficient				Cronbach's Alpha	Factor
**ETLQ**	**CT**	**LA**	**TE**		
0.932	0.436	0.553	1	0.942	TE (19 items)
0.0001	0.0001	0.0001			
0.080	0.639	1		0.893	LA (10 Items)
0.0001	0.0001				
0.645	1			0.756	CT (4 items)
0.0001					
1				0.944	ETLQ

To examine the internal correlation (internal reliability) of modified ETLQ, Cronbach’s alpha was obtained equal to 0.944 for the whole tool (Table 9). Based on internal consistency evaluation for each of the three sections and seven factors of the questionnaire, Cronbach’s alpha was in the 0.756—0.942 range, which means that the subscales have the required reliability (Table 8). As listed, since the *p*-value < 0.05, the correlation between the factors in the model is significant.

**Table 9:** Internal consistency of the Modified ETLQ and its sections and factors in Nursing Students

Factors / section and scale	Cronbach's Alpha	Items No
Relevant and evoke interest	0.888	1,2,3,4,6,7
Alignment	0.843	5,8,10,11,12
Peer Support	0.856	9,13,14,15
Constructive Feedback	0.837	16, 17, 18
Deep approach	0.846	23, 24, 25, 26, 29
Surface approach	0.789	20, 21, 22, 27, 28
Critical learning	0.756	30,31,32,33 4 items
Teaching learning environment	0.893	10 items
Approach to learning	0.942	19 items
Modified ETLQ	0.944	33 items

## Discussion

This study was performed with the aim of collecting validity evidence of the modified experiences of teaching–learning questionnaire (ETLQ) in Iranian nursing students.

At first, cultural validation was performed and showed that the Farsi translation of Modified ETLQ has the content and response process assessment. The results also showed that CVI and CVR were equal to 0.85 and 0.8 respectively, which are normally used for quantitative content validity assessments [18, 19]. The value of CVI and CVR in this study supported quantitative content validity of the tool. To evaluate the status of the response process, the opinions and views of nursing students were examined and their viewpoints were evaluated and modified in terms of concepts, perceptions, grammar, and literature. In many studies, such evaluations are performed on questionnaires using face validity [20].

In the internal structure phase, the results of EFA and CFA in the first part of the modified ETLQ (teaching–learning environment) showed that the model of this section was confirmed with 19 items and four factors (67.5% variance of 19 items) for nursing students. In addition, the model goodness of fit indices also confirmed this (RMSEA = 0.071, NNFI = 0.90, CFI = 0.90, AGFI = 0.81 and X2 / df = 2.5). However, in Utriainen et al., the first part had four factors and the fourth factor was confirmed with three sub-factors [14]. One of the reasons for this discrepancy could be related to the sample size. Perhaps if the sample size had been larger, the results would have been different.

The results of EFA and CFA showed that the second part of the modified ETLQ (students’ approach to learning) in nursing students had 10 items and two factors, which accounted for 60.124% of the variance of 10 items. The goodness of fit indices also confirmed the above model with 10 items and two factors (RMSEA = 0.071, NNFI = 0.91, CFI = 0.91, AGFI = 0.81 and X2 / df = 2.15). Utriainen et al. also confirmed the second part of the Modified ETLQ with 10 items and three subscales [14]. This study used nursing students and a major part of their program was clinical education. Therefore, differences in study participants can affect their approach to learning.

The results of EFA and CFA showed that the third part of the modified ETLQ (critical thinking) in nursing students had four items and the goodness of fit indices confirmed the 4-item one-factor model (RMSEA = 0.032, NNFI = 0.99, CFI = 0.1, AGFI = 0.98 and X2 / df = 1.27) In Utriainen et al., this section also had four items and a single factor [14].

The results of the MKO test supported the presumptions of EFA and the EFA results confirmed construct validity of three factors with specific value > 2; so that 53.49% of the variance of 33 items is attributed to these three factors. Karagiannopoulou and Milienos used EFA and reported that four factors in ETLQ explained 40.2% of variance in 40 items [4]. Utriainen et al. only used CFA and found three factors for 33 items [14]. To explain the findings, the EFA successfully determined the main factors of the scale and the Farsi translation of the tool had a good construct validity for Iranian nursing students.

The CFA results supported the modified ETLQ with 33 items (RMSEA = 0.064, NNFI = 0.93, CFI = 0.94, AGFI = 0.84, X2/df = 2.65). Therefore, the goodness of fit of the indices in the Farsi modified ETLQ for nursing students is supported. Utrianen et al. [14] reported goodness of fit of ETLQ scale (CFI = 0.94, TLI = 0.93, RMSEA = 0.04). Factor analysis by Karagiannopoulou and Milienos [4] indicated the goodness of fit with four factors and 40 items (CFI = 0.92, GFI = 0.9, RMSEA = 0.02). Clearly, the results by other studies are in line with the present study. Therefore, the CFA results supported the construct validity of the Farsi translation of modified ETLQ.

Reliability of the tool based on the test–retest method was 0.88 and Pearson’s correlation indicated a direct and significant relationship between the items and the subscales and the whole ETLQ. This shows the internal consistency of the tool. Cronbach’s alpha of the tool was equal to 0.944 and in the 0.756 – 0.944 range for the subscales, which indicates internal validity of the tool. Karagiannopoulou and Milienos also found a direct and significant correlation between the items and subscales of the tool and the whole tool [4]. To explain the findings, the internal reliability of modified ETLQ for the nursing student population was supported.

The aim of this study was to standardize tools for researchers and educators to be used in processes and educational interactions with nursing students and collecting their valuable experiences in the teaching or learning process. The use of standard tools can provide very useful and reliable results for examining the status and experiences of teaching–learning in students [14]. Therefore, this tool can increase the knowledge and awareness of nursing educators about the teaching–learning experiences of nursing students and help them in planning and using appropriate and effective teaching–learning strategies.

### Study strengths and limitations

The study was conducted from April to November 2020 and because of the limitations of COVID-19 disease, the questionnaires needed for EFA and CFA were distributed only as electronic files. The strength of this study is the use of standard steps for translation and cultural validation, as well as the use of appropriate statistical methods and heuristic and confirmatory factor analysis on two sample groups of different sizes. In addition, to improve the validity and reliability of the instrument in Iranian nursing students, the content, internal structure, and response process were systematically and regularly focused in the process of collecting evidences. Due to the coronary pandemic, the return rate of completed questionnaire was low. In addition, filling out electronic questionnaires is still not very common in Iranian academic and general communities. Nearly 50% of the students answered the questionnaires. This tool can be used for students of different disciplines with different sample sizes.

## Conclusion

In general, the results of the study showed that the Farsi version of modified ETLQ has three sections, seven factors, and 33 items. This tool has sufficient and acceptable evidence in terms of content, response process, reliability, and internal structure. Therefore, the Farsi version of modified ETLQ in Iranian society has acceptable content, reliability, and internal structure and it can be used in studies related to nursing students in Iran.

## Supplementary Information

**Additional file 1: Complementary Table 1.** Percentage of variance and eigenvalues of different factors- ETLQ, section 1. **Complementary Table 2.** Percentage of variance and eigenvalues of different factors- ETLQ, section 2. **Complementary Table 3.** Percentage of variance and eigenvalues of different factors- ETLQ, section 3. **Complementary Table 4.** Percentage of variance and eigenvalues of different factors- ETLQ.

## Data Availability

The datasets used in the study are available from the corresponding author on reasonable request.

## Abbreviations

TLI: Tucker-Lewis index
NFI: Normed fit index
GFI: Goodness of fit index
EFA: Exploratory factor analysis
CFA: Confirmatory factor analysis
RMSEA: Root mean square error of approximation
CVI: Content validity index
CVR: Content validity ratio
ETLQ: Experiences teaching learning questionnaire
KMO: Kaiser Meyer Olkin
PC: Principal components
KUMS: Kermanshah University of Medical Sciences
