# Supplementary material for: Assessment of psychometric properties of the modified experiences of teaching–learning questionnaire in Iranian nursing students
Source: BMC Med Educ. 2022 Apr 25;22:314. doi: 10.1186/s12909-022-03365-z (PMC9037970; doi:10.1186/s12909-022-03365-z)
Supplement: Supplementary file 1 — Additional file 1: Complementary Table 1. Percentage of variance and eigenvalues of different factors- ETLQ, section 1. Complementary Table 2. Percentage of variance and eigenvalues of different factors- ETLQ, section 2. Complementary Table 3. Percentage of variance and eigenvalues of different factors- ETLQ, section 3. Complementary Table 4. Percentage of variance and eigenvalues of different factors- ETLQ. [file 12909_2022_3365_MOESM1_ESM.docx]

| Component | Initial Eigenvalues | | | Extraction Sums of Squared Loadings | | | Rotation Sums of Squared Loadings | | |
| --- | --- | --- | --- | --- | --- | --- | --- | --- | --- |
|  | Total | Variance (%) | Cumulative (%) | Total | Variance (%) | Cumulative (%) | Total | Variance (%) | Cumulative (%) |
| 1 | 8.630 | 45.419 | 45.419 | 8.630 | 45.419 | 45.419 | 3.579 | 18.837 | 18.837 |
| 2 | 1.741 | 9.161 | 54.580 | 1.741 | 9.161 | 54.580 | 3.477 | 18.298 | 37.134 |
| 3 | 1.250 | 6.580 | 61.160 | 1.250 | 6.580 | 61.160 | 2.933 | 15.438 | 52.572 |
| 4 | 1.053 | 5.542 | 66.702 | 1.053 | 5.542 | 66.702 | 2.685 | 14.130 | 66.702 |
| 5 | .741 | 3.899 | 70.601 |  |  |  |  |  |  |
| 6 | .654 | 3.442 | 74.043 |  |  |  |  |  |  |
| 7 | .618 | 3.253 | 77.296 |  |  |  |  |  |  |
| 8 | .566 | 2.981 | 80.276 |  |  |  |  |  |  |
| 9 | .512 | 2.696 | 82.972 |  |  |  |  |  |  |
| 10 | .502 | 2.644 | 85.616 |  |  |  |  |  |  |
| 11 | .437 | 2.298 | 87.914 |  |  |  |  |  |  |
| 12 | .411 | 2.162 | 90.076 |  |  |  |  |  |  |
| 13 | .358 | 1.885 | 91.961 |  |  |  |  |  |  |
| 14 | .328 | 1.729 | 93.690 |  |  |  |  |  |  |
| 15 | .317 | 1.666 | 95.355 |  |  |  |  |  |  |
| 16 | .278 | 1.463 | 96.818 |  |  |  |  |  |  |
| 17 | .241 | 1.267 | 98.085 |  |  |  |  |  |  |
| 18 | .194 | 1.021 | 99.106 |  |  |  |  |  |  |
| 19 | .170 | .894 | 100.000 |  |  |  |  |  |  |

Complementary Table 1. Percentage of variance and eigenvalues of different factors- ETLQ, Section 1

Complementary Table 2. Percentage of variance and eigenvalues of different factors- ETLQ, Section 2

| Component | Initial Eigenvalues | | | Extraction Sums of Squared Loadings | | | Rotation Sums of Squared Loadings | | |
| --- | --- | --- | --- | --- | --- | --- | --- | --- | --- |
|  | Total | Variance (%) | Cumulative (%) | Total | Variance (%) | Cumulative (%) | Total | Variance (%) | Cumulative (%) |
| 1 | 5.253 | 52.529 | 52.529 | 5.253 | 52.529 | 52.529 | 3.264 | 32.642 | 32.642 |
| 2 | .938 | 9.377 | 61.907 | .938 | 9.377 | 61.907 | 2.926 | 29.265 | 61.907 |
| 3 | .881 | 8.810 | 70.716 |  |  |  |  |  |  |
| 4 | .721 | 7.210 | 77.927 |  |  |  |  |  |  |
| 5 | .535 | 5.353 | 83.279 |  |  |  |  |  |  |
| 6 | .455 | 4.552 | 87.831 |  |  |  |  |  |  |
| 7 | .368 | 3.682 | 91.513 |  |  |  |  |  |  |
| 8 | .323 | 3.235 | 94.747 |  |  |  |  |  |  |
| 9 | .271 | 2.706 | 97.454 |  |  |  |  |  |  |
| 10 | .255 | 2.546 | 100.000 |  |  |  |  |  |  |

Complementary Table 3. Percentage of variance and eigenvalues of different factors- ETLQ, Section 3

| Component | Initial Eigenvalues | | | Extraction Sums of Squared Loadings | | |
| --- | --- | --- | --- | --- | --- | --- |
|  | Total | Variance (%) | Cumulative (%) | Total | Variance (%) | Cumulative (%) |
| 1 | 2.468 | 61.700 | 61.700 | 2.468 | 61.700 | 61.700 |
| 2 | .732 | 18.290 | 79.989 |  |  |  |
| 3 | .491 | 12.267 | 92.257 |  |  |  |
| 4 | .310 | 7.743 | 100.000 |  |  |  |

Complementary Table 4. Percentage of variance and eigenvalues of different factors- ETLQ

| Component | Initial Eigenvalues | | | Extraction Sums of Squared Loadings | | | Rotation Sums of Squared Loadings | | |
| --- | --- | --- | --- | --- | --- | --- | --- | --- | --- |
|  | Total | Variance (%) | Cumulative (%) | Total | Variance (%) | Cumulative (%) | Total | Variance (%) | Cumulative (%) |
| 1 | 12.606 | 38.201 | 38.201 | 12.606 | 38.201 | 38.201 | 7.567 | 22.931 | 22.931 |
| 2 | 3.140 | 9.516 | 47.717 | 3.140 | 9.516 | 47.717 | 5.733 | 17.373 | 40.304 |
| 3 | 1.776 | 5.383 | 53.100 | 1.776 | 5.383 | 53.100 | 4.223 | 12.796 | 53.100 |
| 4 | 1.702 | 5.157 | 58.256 |  |  |  |  |  |  |
| 5 | 1.481 | 4.487 | 62.743 |  |  |  |  |  |  |
| 6 | 1.072 | 3.247 | 65.991 |  |  |  |  |  |  |
| 7 | 1.035 | 3.136 | 69.126 |  |  |  |  |  |  |
| 8 | .867 | 2.627 | 71.753 |  |  |  |  |  |  |
| 9 | .799 | 2.421 | 74.174 |  |  |  |  |  |  |
| 10 | .685 | 2.077 | 76.251 |  |  |  |  |  |  |
| 11 | .647 | 1.961 | 78.212 |  |  |  |  |  |  |
| 12 | .611 | 1.852 | 80.063 |  |  |  |  |  |  |
| 13 | .576 | 1.746 | 81.809 |  |  |  |  |  |  |
| 14 | .566 | 1.715 | 83.524 |  |  |  |  |  |  |
| 15 | .552 | 1.672 | 85.197 |  |  |  |  |  |  |
| 16 | .491 | 1.489 | 86.685 |  |  |  |  |  |  |
| 17 | .463 | 1.403 | 88.088 |  |  |  |  |  |  |
| 18 | .449 | 1.361 | 89.449 |  |  |  |  |  |  |
| 19 | .394 | 1.193 | 90.643 |  |  |  |  |  |  |
| 20 | .370 | 1.120 | 91.763 |  |  |  |  |  |  |
| 21 | .339 | 1.028 | 92.790 |  |  |  |  |  |  |
| 22 | .326 | .989 | 93.779 |  |  |  |  |  |  |
| 23 | .306 | .927 | 94.706 |  |  |  |  |  |  |
| 24 | .274 | .829 | 95.535 |  |  |  |  |  |  |
| 25 | .257 | .779 | 96.314 |  |  |  |  |  |  |
| 26 | .230 | .698 | 97.012 |  |  |  |  |  |  |
| 27 | .186 | .565 | 97.577 |  |  |  |  |  |  |
| 28 | .163 | .494 | 98.071 |  |  |  |  |  |  |
| 29 | .158 | .478 | 98.549 |  |  |  |  |  |  |
| 30 | .144 | .436 | 98.985 |  |  |  |  |  |  |
| 31 | .137 | .414 | 99.399 |  |  |  |  |  |  |
| 32 | .126 | .381 | 99.781 |  |  |  |  |  |  |
| 33 | .072 | .219 | 100.000 |  |  |  |  |  |  |
